# Optimizing Thermoplastic Starch Film with Heteroscedastic Gaussian Processes in Bayesian Experimental Design Framework

**DOI:** 10.3390/ma17215345

**Published:** 2024-10-31

**Authors:** Gracie M. White, Amanda P. Siegel, Andres Tovar

**Affiliations:** 1 Luddy School of Informatics, Computing, and Engineering, Integrative Nanosystems Development Institute (INDI),} Indiana University Indianapolis, Indianapolis, IN 46202, USA; whitegra@iu.edu; 2 Department of Chemistry and Chemical Biology, Indiana University Indianapolis, Indianapolis, IN 46202, USA; apsiegel@iu.edu; 3 School of Mechanical Engineering, Purdue University, Indianapolis, IN 46202, USA

**Keywords:** thermoplastic starch, potato starch, compostable plastic, Bayesian optimization, heteroscedastic noise, material design, physical experiments, mechanical properties

## Abstract

The development of thermoplastic starch (TPS) films is crucial for fabricating sustainable and compostable plastics with desirable mechanical properties. However, traditional design of experiments (DOE) methods used in TPS development are often inefficient. They require extensive time and resources while frequently failing to identify optimal material formulations. As an alternative, adaptive experimental design methods based on Bayesian optimization (BO) principles have been recently proposed to streamline material development by iteratively refining experiments based on prior results. However, most implementations are not suited to manage the heteroscedastic noise inherently present in physical experiments. This work introduces a heteroscedastic Gaussian process (HGP) model within the BO framework to account for varying levels of uncertainty in the data, improve the accuracy of the predictions, and increase the overall experimental efficiency. The aim is to find the optimal TPS film composition that maximizes its elongation at break and tensile strength. To demonstrate the effectiveness of this approach, TPS films were prepared by mixing potato starch, distilled water, glycerol as a plasticizer, and acetic acid as a catalyst. After gelation, the mixture was degassed via centrifugation and molded into films, which were dried at room temperature. Tensile tests were conducted according to ASTM D638 standards. After five iterations and 30 experiments, the films containing 4.5 wt% plasticizer and 2.0 wt% starch exhibited the highest elongation at break (M = 96.7%, SD = 5.6%), while the films with 0.5 wt% plasticizer and 7.0 wt% starch demonstrated the highest tensile strength (M = 2.77 MPa, SD = 1.54 MPa). These results demonstrate the potential of the HGP model within a BO framework to improve material development efficiency and performance in TPS film and other potential material formulations.

## 1. Introduction

The growing need to reduce plastic environmental pollution [1] and mitigate microplastic health risks [2] demands sustainable alternatives to traditional fossil-based plastics. One alternative is thermoplastic starch (TPS), a biodegradable material derived from natural polymers [3]. TPS has been used in applications like food packaging [4,5] and as a substitute for single-use plastics [6]. However, its mechanical properties are considerably inferior to those of fossil-based plastics [7]. For example, film-grade high-density polyethylene (HDPE), a fossil-based plastic commonly used in packaging, exhibits an elongation at break (EB) between 350% and 1700% and tensile strength (TS) ranging from 23.0 MPa to 89.2 MPa [8]. In contrast, typical TPS film shows EB values around 11% to 31%, and TS values around 1.03 MPa to 2.04 MPa [9,10].

Although the performance of TPS can be enhanced with additives such as Aloe vera [9], chitin nanocrystals [11], zinc oxide [12], nanoclay [13], agar [14], psyllium husk [15], and different plasticizers [10,16,17,18], the development and optimization of new compositions is a lengthy and expensive process that typically involves trial-and-error and design of experiments (DOE) methods. While DOE methods such as grid searching and response surface can help us understand the effect of material parameters on its properties, they are often resource-intensive when used for optimization due to the large number of design evaluations required [19]. Experimental noise further complicates the process, especially when time and resource constraints prevent accurate uncertainty quantification. To overcome these challenges, adaptive experimental methods supported by BO are becoming increasingly prevalent as they offer more efficient solutions than traditional DOE methods.

### 1.1. Bayesian Experimental Design

Bayesian optimization (BO) is a gradient-free, global optimization method suitable for expensive-to-evaluate functions. It uses a probabilistic model, typically a Gaussian process (GP), to predict the function mean and variance (Section 2). It balances the exploration and exploitation of the design space via an acquisition function that selects the next design while minimizing the number of costly and time-consuming experiments (Section 3). In material design, BO offers a probabilistic framework that iteratively plans experiments based on prior results. This enables a more informed and efficient experimental process that guides decision-makers toward a globally optimal design [20,21,22].

In recent years, BO has gained attention in optimal material design and fabrication for its ability to efficiently navigate high-dimensional search spaces and identify optimal formulations and process parameters with fewer experimental evaluations [23,24]. Packwood [25] optimized the processing parameters of metal alloys and polymer blends, focusing on tuning processing conditions like temperature, pressure, and material composition. Yamashita et al. [26] optimized material atomic configurations, such as crystal, surface, and interface structures, and predicted the most stable structure for given chemical compositions. Talapatra et al. [27] introduced Bayesian objective under model uncertainty (BOMU) to explore the materials design space, accounting for resource constraints and model uncertainty. Liu et al. [28] introduced a BO approach for categorical and non-categorical variables for multimaterial vehicle structures. Xiong et al. [29] improved the mechanical properties of metals for additive manufacturing. Zhang et al. [30] accommodated mixed quantitative and qualitative variables and efficiently identified materials with desired dielectric characteristics. Zhang et al. [31] enhanced the precision and material properties in additive manufacturing, focusing on parameters like layer thickness, build speed, and material feed rate. Gao et al. [32] identified monomers and fabrication conditions of membranes for water purification. Iwama et al. [33] applied a Bayesian adaptive experimental design to optimize the operating conditions and reaction parameters in an ethylene oxide production plant. Valladares et al. [34] introduced a goal-based acquisition function that enables the parallel Bayesian optimization of lithium-ion battery cathode composition. Hickman et al. [35] fine-tuned reaction parameters such as temperature, pressure, and catalyst concentration to maximize reaction yield and selectivity. Guo et al. [36] improved high-throughput reaction screening by optimizing multiple reaction parameters simultaneously, allowing for faster identification of optimal CO_2_ and green hydrogen production conditions. Qian et al. [37] leverage BO with the mean objective cost of uncertainty (MOCU) to optimize functional materials. Sattari et al. [38] incorporated physical constraints and process knowledge to improve process precision and mechanical performance of additively manufactured parts.

Although Bayesian optimization is emerging as a valuable tool for material design and has been successfully applied to optimize various material systems, its application in physical experiments is still in the early stages of development, with several challenges yet to be addressed. The challenges addressed in this work include the following: (1) Modeling heteroscedastic noise in physical experiments; (2) The adaptive exploration of the design space; (3) The application toward locating optimal TPS film formulations.

### 1.2. Heteroscedastic Noise

In most adaptive material design applications, the GP regression model supporting BO captures the underlying relationships between experimental parameters (design variables) and the material properties (objective function). However, traditional GP models assume that noise levels are uniformly distributed across the design space [39]. Since noise levels are not uniform across the design space in many real-world situations, the homeostatic noise assumption presents a challenge in experimental research, specifically relating to the field of materials science [40]. Heteroscedastic noise, which refers to variable levels of uncertainty across the design space, can significantly affect experimental outcomes and data interpretation. This can lead to inaccurate uncertainty quantification and statistical outcomes. Uncertainty in observations may even follow a random distribution, making heteroscedastic inferences analytically intractable [41].

Previous work has yielded methods to approximate the posterior noise variance using heteroscedastic Gaussian process (HGP) interfaces, with the gold standard being the Markov chain Monte Carlo (MCMC) method [42]; however, methods such as MCMC come at a high cost since they require many additional experimental design evaluations and time to evaluate [43]. Therefore, a practical HGP model is needed for the adaptive experimental design of materials within a BO framework.

The first contribution of this work is introducing a practical HGP model suitable for Bayesian experimental design (Section 2.3). The proposed HGP model uses two uncoupled GPs: one to model the mean of the material properties and one to model the variance (noise). Since the heteroscedastic noise function is modeled independently from the material properties, the mean GP model does not require prior noise information, making its implementation practical and efficient. The four statistical outcomes (two per GP) provide a simple and accurate way to estimate the expected value of the material properties and quantify their uncertainty.

### 1.3. Adaptive Exploration

Most BO implementations for physical experiments follow the same general steps as the ones used for computational experiments. In this configuration, an acquisition function identifies one promising design to be evaluated at a time based on the outputs of the GP (mean and variance). In a physical setting, evaluating one design per iteration causes experiments to advance unnecessarily slowly, especially considering situations where individual designs are timely and expensive to evaluate. Since many physical experiments inherently require time-consuming setups, considering more than one design candidate per iteration is manageable and more efficient. Therefore, there is a need for an approach to identify multiple candidate designs systematically.

Additionally, most implementations use fixed design space boundaries; however, too large boundaries can lead to unfeasible material designs, while boundaries that are too small may leave important regions unexplored. Thus, the boundaries must be adapted as the experiment progresses, allowing the exploration region to be refined based on prior experimental results.

The second contribution of this work is the application of two acquisition functions while managing the size of the exploration within a BO framework. The two acquisition functions are the lower confidence bound (LCB) and expected improvement (EI). The LCB function allows for increased diversity of new design candidates by promoting more exploration or exploitation than the traditional EI (Section 3.1). In addition, an exploration region management algorithm is implemented to dynamically adjust the size of the exploration region based on the inclusion of new designs in the sampling plan to ensure a more comprehensive modeling approach.

### 1.4. Optimal Formulation of TPS Film

BO has been applied successfully to optimize various material systems, including metal alloys, crystal structures, battery cathodes, and polymer blends. This work presents the first application of BO for formulating TPS films. The specific objective of this work is to determine the optimal starch and plasticizer weight percentages (wt%) in the composition of TPS films to maximize elongation at break and tensile strength (Section 4). This marks the first use of BO in the design of TPS films.

In summary, the three main contributions of this work are as follows: (1) The introduction of a practical HGP model suitable for Bayesian experimental design; (2) An adaptive exploration of the design space based on acquisition functions and exploration region management; (3) The optimal TPS film formulation for the maximization of elongation at break and tensile strength. This work shows the potential of Bayesian experimental design in advancing the development of sustainable materials, with a particular focus on TPS films. By leveraging HGP modeling and adaptive exploration, this work contributes to the broader field of material science and offers a blueprint for future research in optimizing renewable materials.

## 2. Gaussian Process Regression

A Gaussian process (GP) is a collection of random variables such that every finite set of those variables has a multivariate Gaussian distribution. In the context of regression, a GP regression model can be defined as a distribution over functions with inference taking place directly in the space of functions [44]. A GP regression model defines a distribution of functions f(x) such that, for any set of input points $X=\{x_{1},\ldots ,x_{n}\}$, the function values $f=f\left(X\right)=\{f\left(x_{1}\right),\ldots ,f\left(x_{n}\right)\}$ follow a multivariate normal distribution [45]. Unlike traditional regression techniques that assume a specific functional form for the relationship, a GP regression model defines a distribution over possible functions that fit the data, allowing for a more adaptable and probabilistic modeling approach.

### 2.1. Gaussian Process Regression of Noiseless Data

A Gaussian process (GP) is fully specified by its mean function and covariance function (kernel). A random function f(x) that follows a GP is expressed as

(1) $$f\left(x\right)∼\mathrm{GP}\left(m\left(x\right),k\left(x,x^{\prime }\right)\right)\;,$$

where m(x) is the mean function and $k(x,x^{\prime })$ is the covariance function defined as

(2) $$\begin{array}{cc} m(x) & =E[f(x)]\;, \end{array}$$

(3) $$\begin{array}{cc} k(x,x^{\prime }) & =E[\left(f\left(x\right)-m\left(x\right)\right)\left(f\left(x^{\prime }\right)-m\left(x^{\prime }\right)\right)]\;, \end{array}$$

respectively. The joint prior distribution of the observations $f=\{f\left(x_{1}\right),\ldots ,f\left(x_{n}\right)\}$ and the predictions $f_{*}$ at the test locations $X_{*}$ is

(4) $$\left[\begin{array}{c} f\\ f_{*} \end{array}\right]∼N\left(\begin{array}{c} 0,\left[\begin{array}{cc} K(X,X) & K(X,X_{*})\\ K(X_{*},X) & K(X_{*},X_{*}) \end{array}\right] \end{array}\right),$$

where K(X,X) is the covariance matrix of the observed data, $K(X,X_{*})$ is the covariance matrix between the observed data and the predicted data, and $K(X_{*},X_{*})$ is the covariance matrix of the predictions. The components of the covariance matrices are generated by the evaluation of the covariance function $k(x,x^{\prime })$. Valid covariance functions produce positive semi-definite matrices regardless of the chosen pair of points $\left(x,x^{\prime }\right)$ [46].

### 2.2. Gaussian Process Regression of Noisy Data

In modeling problems involving experimental observations, the data do not correspond to observations of f(x), but noisy values, y=f(x)+ϵ, where $\epsilon ∼N(0,\sigma_{n}^{2})$. Given a set of noisy observations, $y=\{y^{\left(1\right)},\ldots ,y^{\left(n\right)}\}$, the joint prior distribution of y and the predictions $f_{*}$ at the test locations $X_{*}$ is   

(5) $$\left[\begin{array}{c} y\\ f_{*} \end{array}\right]∼N\left(\begin{array}{c} 0,\left[\begin{array}{cc} K\left(X,X\right)+\sigma_{n}^{2}I & K(X,X_{*})\\ K(X_{*},X) & K(X_{*},X_{*}) \end{array}\right] \end{array}\right)\;,$$

where $\sigma_{n}^{2}$ is the noise in the data (under the assumption of independent Gaussian noise), and I is the identity matrix.

In most cases, the covariance function $k(x,x^{\prime })$ is flexible enough to fit the data, and the mean function is often assumed to be zero, m(x)=0 [47]. Then, the predictive equations of a zero-mean GP regression model are obtained by conditioning Equation (5) on the observed data by setting the following condition:

(6) $$f_{*}|X,y,X_{*}∼N\left({\bar{f}}_{*},\mathrm{cov}\left(f_{*}\right)\right).$$

where ${\bar{f}}_{*}$ and $\mathrm{cov}(f_{*})$ are the mean and the covariance of the prediction, which are given by the posterior distribution mean and variance:

(7) $$\begin{array}{cc} {\bar{f}}_{*} & =K\left(X_{*},X\right){\left[K\left(X,X\right)+\sigma_{n}^{2}I\right]}^{-1}y, \end{array}$$

(8) $$\begin{array}{cc} \mathrm{cov}(f_{*}) & =K\left(X_{*},X_{*}\right)-K\left(X_{*},X\right){\left[K\left(X,X\right)+\sigma_{n}^{2}I\right]}^{-1}K\left(X,X_{*}\right). \end{array}$$

where the diagonal of $\mathrm{cov}(f_{*})$ is the predictive variance [44]. Since the mean function becomes irrelevant, the covariance function (kernel) defines the characteristics of the prediction. Notably, the covariance prediction does not require the observations y because it is a property of the GP model and the distribution of the inputs only, regardless of the observed values.

### 2.3. Heteroscedastic Gaussian Process Regression

Heteroscedastic Gaussian Process (HGP) regression is an extension of traditional GP regression that deals with situations where the noise $\epsilon ∼N(0,\sigma_{n}^{2})$ is not constant across the input space. While standard GP regression assumes a constant $\sigma_{n}^{2}$ across all inputs, HGP regression allows noise variance to change depending on the input, making it more flexible and accurate for physical world scenarios where uncertainty is not uniform.

The model is structured in HGP regression with two main components: a mean function and a variance function drawn from different Gaussian processes. This is shown below:

(9) $$\begin{array}{cc} f_{1}\left(x\right) & ∼\mathrm{GP}\left(0,k_{1}\left(x,x^{\prime }\right)\right) \end{array}$$

(10) $$\begin{array}{cc} f_{2}\left(x\right) & ∼\mathrm{GP}\left(0,k_{2}\left(x,x^{\prime }\right)\right)\;. \end{array}$$

where $f_{1}$ follows a mean function GP that captures the underlying relationship between inputs x and outputs y, while $f_{2}$ follows the noise variance function GP that captures the relationship between inputs x and the noise variance $\sigma_{n}^{2}$. In this work, the HGP model outputs are given by the following:

(11) $$\begin{array}{cc} {\bar{f}}_{1*} & =K\left(X_{*},X\right)K{\left(X,X\right)}^{-1}y \end{array}$$

(12) $$\begin{array}{cc} \mathrm{cov}(f_{1*}) & =K\left(X_{*},X_{*}\right)-K\left(X_{*},X\right)K{\left(X,X\right)}^{-1}K\left(X,X_{*}\right)+\mathrm{diag}\left(f_{2*}\right)+\mathrm{cov}\left(f_{2*}\right) \end{array}$$

(13) $$\begin{array}{cc} {\bar{f}}_{2*} & =K\left(X_{*},X\right){\left[K\left(X,X\right)+\lambda I\right]}^{-1}\sigma_{n}^{2} \end{array}$$

(14) $$\begin{array}{cc} \mathrm{cov}(f_{2*}) & =K\left(X_{*},X_{*}\right)-K\left(X_{*},X\right){\left[K\left(X,X\right)+\lambda I\right]}^{-1}K\left(X,X_{*}\right)\;. \end{array}$$

where ${\bar{f}}_{1*}$ and $\mathrm{cov}(f_{1*})$ are the mean and the covariance of the function prediction, ${\bar{f}}_{2*}$ and $\mathrm{cov}(f_{2*})$ are the mean and the covariance of the noise variance, and diag(f) is the diagonal matrix constructed from the vector f. Here, $\sigma_{n}^{2}$ is the noise variance, and λ is a trainable hyperparameter representing its variance—the variance of the noise variance.

### 2.4. Covariance Function

The covariance function $k(x,x^{\prime })$ encodes assumptions about the smoothness, periodicity, and other properties of the function to be modeled. The choice of covariance function is crucial in GP regression as it defines the shape and properties of the functions that the GP can model. Commonly used covariance functions include the squared exponential kernel, the Matérn kernel, and the periodic kernel.

The squared exponential kernel, also referred to as the radial basis function (RBF), is defined as

(15) $$k\left(x,x^{\prime }\right)=\sigma_{f}^{2}\exp\left(-\frac{∥x-x^{\prime }{∥}^{2}}{2\ell^{2}}\right)\;.$$

where $\sigma_{f}^{2}$ is the signal variance, and *ℓ* is the length scale, controlling the smoothness of the function. Similarly, the Matérn kernel is given by

(16) $$k\left(x,x^{\prime }\right)=\sigma_{f}^{2}\;\frac{2^{1-\nu }}{\Gamma (\nu )}{\left(\frac{\sqrt{2\nu }∥x-x^{\prime }∥}{\ell }\right)}^{\nu }K_{\nu }\left(\frac{\sqrt{2\nu }∥x-x^{\prime }∥}{\ell }\right)\;.$$

where $K_{\nu }\left(\right)$ is a modified Bessel function and Γ(ν) is the Gamma function [44]: for a positive integer ν, Γ(ν)=(ν−1)!. Here, ν controls the smoothness, and the Gamma function Γ(ν) normalizes the kernel function and ensures that it correctly describes the covariance structure of the process. The Matérn covariance function becomes simpler when ν is a half-integer, such as 1/2, 3/2, 5/2, 7/2, and so on. For ν=1/2, the process becomes very rough, while ν≥7/2 may be indistinguishable from ν→∞[44]. The most common cases in GP regression are ν=3/2 and ν=5/2, resulting in

(17) $$\begin{array}{cc} k_{v=3/2}\left(x,x^{\prime }\right) & =\sigma_{f}^{2}\;\left(1+\frac{\sqrt{3}∥x-x^{\prime }∥}{\ell }\right)\exp\left(-\frac{\sqrt{3}∥x-x^{\prime }∥}{\ell }\right)\;, \end{array}$$

(18) $$\begin{array}{cc} k_{v=5/2}\left(x,x^{\prime }\right) & =\sigma_{f}^{2}\;\left(1+\frac{\sqrt{5}∥x-x^{\prime }∥}{\ell }+\frac{5∥x-x^{\prime }{∥}^{2}}{3\ell^{2}}\right)\exp\left(-\frac{\sqrt{5}∥x-x^{\prime }∥}{\ell }\right)\;, \end{array}$$

respectively. Finally, a periodic kernel can be defined as

(19) $$k\left(x,x^{\prime }\right)=\sigma_{f}^{2}\exp\left(-\frac{2\sin^{2}(\pi ∥x-x^{\prime }∥/p)}{\ell^{2}}\right)\;.$$

where *p* is the period of the function.

### 2.5. Training

Training involves finding the optimal values of the unknown kernel parameters, such as $\sigma_{f}^{2}$, *ℓ*, and *p*, which is crucial for achieving good model performance. This task can be accomplished by maximizing the likelihood of the observed data,

(20) $$L\left(\theta |y\right)=p\left(y|X,\theta \right)=N(y|0,K\left(X,X;\theta \right)+\sigma_{n}^{2}I)\;,$$

where K(X,X;θ) is the covariance matrix defined by the kernel function with hyperparameters θ. To facilitate the optimization process, the log-likelihood is often used instead of the likelihood, Equation (20) [44]. This log-likelihood is given by

(21) $$\log p\left(y|X,\theta \right)=-\frac{1}{2}y^{T}{\left(K\left(X,X;\theta \right)+\sigma_{n}^{2}I\right)}^{-1}y-\frac{1}{2}\log\left|K\left(X,X;\theta \right)+\sigma_{n}^{2}I\right|-\frac{n}{2}\log2\pi \;.$$

This function can be maximized using a gradient-based algorithm such as L-BFGS-B (limited-memory Broyden–Fletcher–Goldfarb–Shanno with box constraints). The implementation in this work uses the Python library gpflow version 2.9.1 (https://www.gpflow.org/, accessed on 10 September 2024).

### 2.6. Heteroscedastic Gaussian Process Regression Algorithm

The steps involved in generating the posterior mean and variance of a given set of observed data from a noisy (unknown) function are the following:

Step 1: Input points. Define the input points $X=\{x_{1},\ldots ,x_{n}\}$ using a sampling plan. This set of points can be selected from a predefined grid, a traditional DOE, or quasi-randomly.

Step 2: Observations. Evaluate the noisy function at every $x_{i}$ a given number of times to obtain the corresponding observations $y_{i}$. For example, one can evaluate the noisy function five times at each input point.

Step 3: HGP Regression. At each input point, obtain the observed data’s mean and variance. Use these values to obtain the posterior mean and variance as defined by (11) and (12).

### 2.7. Numerical Example

Let us consider a noisy version of Forrester’s test function defined by

(22) $$f\left(x\right)={\left(6x-2\right)}^{2}\sin\left(12x-4\right)+\epsilon \left(x\right)\;,$$

defined in the interval x∈[0,1] with heteroscedastic Gaussian noise $\epsilon \left(x\right)∼N(0,\sigma^{2}\left(x\right))$ with variance

(23) $$\sigma^{2}\left(x\right)=10{\left(x-0.3\right)}^{2}\;.$$

Let us consider the sampling plan

**Figure 1:**
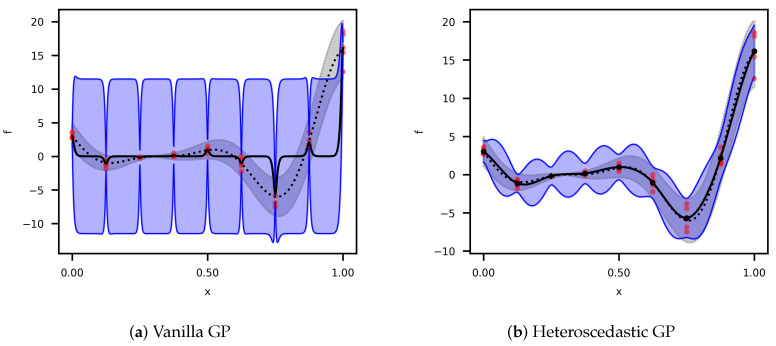
Gaussian process regressions of the noisy Forrester’s function. The red dots represent the random samples. The dotted line and gray-shaded area correspond to the 95% confidence interval. The black line and blue-shaded area correspond to the prediction based on the posterior distributions for the following: (**a**) The vanilla GP; (**b**) The HGP.

X=[0,1/8,1/4,3/8,1/2,5/8,3/4,7/8,1],

with five random observations per point. Figure 1 shows the comparison of the vanilla GP regression from (7) and (8), and the HGP regression from (11) and (12). Both models utilized the Matérn 3/2 kernel (17). Since the vanilla GP cannot capture the noise variance, it fits a constant value across the design space, increasing the prediction’s uncertainty. The predicted mean significantly differs from the true mean of the function. On the other hand, the HGP better fits both the variance and the mean of the true function.

## 3. Bayesian Optimization

Bayesian optimization (BO) is an efficient method for optimizing expensive black-box functions [21,22]. Unlike traditional optimization techniques, which often rely on gradient information or extensive sampling of the parameter space, BO leverages a probabilistic model to guide the search for optimal solutions. This approach is particularly well-suited for applications where each function evaluation is costly in terms of time or resources [48].

In this work, BO begins by modeling the noisy objective function f(x) using a HGP regression (Section 2.6). The HGP predicts the posterior mean (11) and variance (12) of f(x). Then, an acquisition function is defined to determine the next points to sample based on the HGP outcomes (Section 3.1). The function is evaluated in the new points, the HGP model is updated, and this process continues until no improvement is possible. The next sections explain in more detail the acquisition functions implemented in this work, the exploration region management, and the convergence criteria of the BO algorithm.

### 3.1. Acquisition Functions

This work implements two acquisition functions: lower confidence bound (LCB) and the expected improvement (EI). Both functions are designed to balance exploration and exploitation by considering the GP model’s predicted mean function μ(x) defined from (11) and the predicted standard deviation σ(x) obtained from (12).

**Lower Confidence Bound.** The Lower Confidence Bound (LCB) is an acquisition function defined for minimizing a function f(x). The LCB for a given input x is defined by:

(24) LCB(x)=μ(x)−κσ(x),

where κ is a positive parameter that controls the trade-off between exploration (κ→∞) and exploitation (κ=0). If the objective is to minimize f(x), then the new point to be added to the sampling plan is

(25) $$x_{\mathrm{new}}=\arg\min\mathrm{LCB}\left(x\right)\;.$$

In the proposed algorithm, the value of κ is incrementally increased until $x_{\mathrm{new}}$ is different than the one from maximizing the expected improvement.

**Expected Improvement.** Given the mean best-observed value of the function so far $f(x_{\mathrm{best}})$ of the function f(x) to be minimized, the Expected Improvement (EI) at a given point x is defined as:

(26) $$\mathrm{EI}\left(x\right)=E[\max\left(0,f\left(x\right)-f\left(x_{\mathrm{best}}\right)\right)]\;.$$

When the function f(x) is modeled as a GP, the EI function can be expressed as:

(27) $$\mathrm{EI}\left(x\right)=\left(\mu \left(x\right)-f\left(x_{\mathrm{best}}\right)\right)\Phi \left(\frac{\mu \left(x\right)-f\left(x_{\mathrm{best}}\right)}{\sigma (x)}\right)+\sigma \left(x\right)\phi \left(\frac{\mu \left(x\right)-f\left(x_{\mathrm{best}}\right)}{\sigma (x)}\right)\;,$$

where Φ(·) is the cumulative distribution function (CDF) of the standard normal distribution, and ϕ(·) is the probability density function (PDF) of the standard normal distribution. Then, the new point to be added to the sampling plan is

(28) $$x_{\mathrm{new}}=\arg\max\mathrm{EI}\left(x\right)\;.$$

### 3.2. Exploration Region Management

The exploration region is defined as

(29) $$\Gamma =\{x\in R^{n}\;|\;x_{\mathrm{LB}}\leq x\leq x_{\mathrm{UB}}\}\;,$$

where $x_{\mathrm{LB}}$ and $x_{\mathrm{UB}}$ are the lower and upper boundaries, respectively. The boundaries are defined as a function of the distance between the designs x in Γ along every dimension k=1,…,n. This is

(30) $$x_{k}^{\mathrm{UB}}-x_{k}^{\mathrm{LB}}=r\left(x_{k}^{\max}-x_{k}^{\min}\right)\;,$$

where $(x_{k}^{\max}$ and $x_{k}^{\min}$ are the maximum and minimum values along the dimension *k* and r>1 is the expansion ratio. In this work, r=1.5 by default. The corners of the exploration region Γ are also added to the sampling plan. Hence, the exploration grows by a factor *r* in every iteration until it reaches absolute limits, such as a fraction $x_{k}=0$ or $x_{k}=1$ of a given ingredient $x_{k}$.

### 3.3. Convergence Criterion

Soft convergence is achieved when there is no significant change in the value of maxEI(x) and $x_{\mathrm{best}}$ in three consecutive iterations. This is shown below:

(31) $$\begin{array}{cc} \Delta^{3}\max\mathrm{EI}\left(x\right) & \leq \epsilon_{e} \end{array}$$

(32) $$\begin{array}{cc} \Delta^{3}x_{\mathrm{best}} & \leq \epsilon_{x}\;, \end{array}$$

where $\epsilon_{e}$ and $\epsilon_{x}$ are small quantities. Hard convergence occurs when the allotted number of experiments has been exhausted.

### 3.4. Bayesian Optimization Algorithm

**Figure 2:**
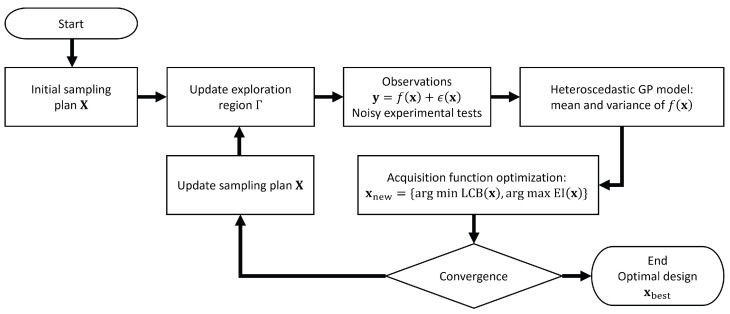
BO adaptive experimental design algorithm implementing an HGP surrogate model and an exploration region.

The steps involved in the proposed Bayesian optimization (BO) approach are summarized in (Figure 2) and include the following steps:

Step 1: Objective Function Regression. BO begins by modeling the objective function f(x) using a HGP regression. The HGP predicts the posterior mean (11) and variance (12) of the noisy objective function f(x) as a distribution over possible functions that fit the observed noisy data y from the sampling plan X.

Step 2: Acquisition Function Optimization. An acquisition function is defined to determine the next point to sample based on the HGP posterior mean and variance. In this work, the new points to be added to the sampling plan correspond to the solutions of (25) and (28).

Step 3: Check Convergence. If the convergence conditions (31) and (32) are satisfied, and the exploration region Γ has reached its maximum size, then the argminμ(x) is the optimal design $x^{*}$. Otherwise, the process continues by adding $x_{\mathrm{new}}$ to the sampling plan.

### 3.5. Numerical Results

To illustrate the implementation of the BO algorithm, let us consider two benchmark problems: Forrester’s function (1D) and the three-hump camel function (2D).

**Figure 3:**
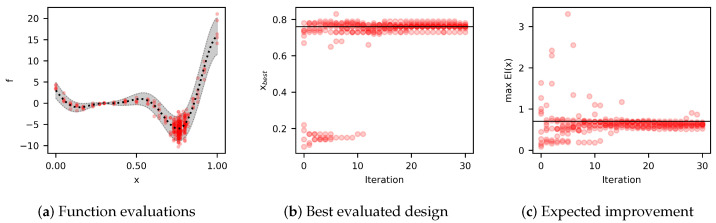
Bayesian optimization of the noisy Forrester’s function. Results summarize ten optimization algorithm runs. Each optimization algorithm was initialized with five random initial designs and ran for 30 iterations. The red dots represent the designs evaluated during all the optimization runs. (**a**) The black dotted line is the mean of the true function, and the gray shaded area is the 95% confidence interval. Most of the function evaluations were around the minimizer. (**b**) The algorithm usually finds the minimizer or a close design in less than five iterations. The black solid line represents the minimizer $x^{*}\approx 0.7572$. (**c**) Accordingly, the expected improvement also remains at a constant value after the fifth iteration.

**Forrester’s function (1D).** The first problem is the minimization of the noisy Forrester’s function defined in (22). The global minimizer of this function is $x^{*}\approx 0.7572$. For this study, let us consider random initial sampling plans with five random designs. To replicate typical physical experiments, the data are defined as

$$X=\{x_{r}-2\delta ,x_{r}-\delta ,x_{r},x_{r}+\delta ,x_{r}+2\delta \}\;,$$

where $x_{r}$ and δ are random numbers such that all *x* in X is in the interval [0,1]. The minimum distance between adjacent designs is set to 0.01. The results are summarized in Figure 3. These results show that, in most cases, convergence can be achieved in about five iterations.

**Three-hump camel function (2D).** The second problem is the minimization of the noisy three-hump camel function:

(33) $$f\left(x_{1},x_{2}\right)=2x_{1}^{2}-1.05x_{1}^{4}+\frac{x_{1}^{6}}{6}+x_{1}x_{2}+x_{2}^{2}+\epsilon \left(x_{1},x_{2}\right)\;,$$

defined in the region $-2\leq x_{1}\leq 2$ and $-2\leq x_{2}\leq 2$, with heteroscedastic Gaussian noise $\epsilon \left(x_{1},x_{2}\right)∼N\left(0,\sigma^{2}\left(x_{1},x_{2}\right)\right)$ with variance

(34) $$\sigma^{2}\left(x_{1},x_{2}\right)=\frac{1}{50}\left[{\left(x_{1}+1\right)}^{2}+{\left(x_{2}+1\right)}^{2}\right]\;.$$

**Figure 4:**
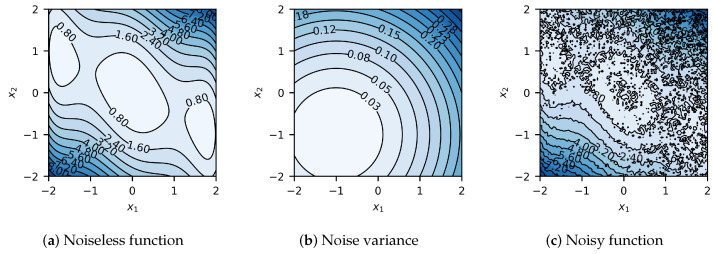
Three-hump camel function prior to optimization. Heteroscedastic noise and variance are to be added to the black box function to simulate physical experimental conditions. (**a**) Noiseless three-hump camel function. (**b**) Heteroscedastic noise variance of the three-hump camel function. (**c**) Noisy three-hump camel function to be optimized.

**Figure 5:**
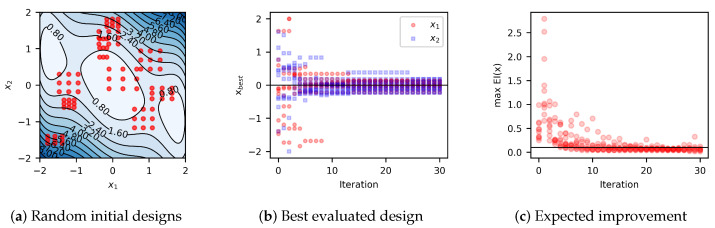
Bayesian optimization of the noisy three-hump camel function. Results summarize ten optimization algorithm runs utilizing an exploration region for each model iteration. Each algorithm was initialized with nine random design points and ran for 30 iterations. The red dots represent the random initial designs. (**a**) The initial designs prior to noisy observations. Most of the initial function evaluations were around local minima, with only one near the minimizer. (**b**) With noisy observations in two dimensions, the algorithm usually finds the minimizer or a close design in less than ten iterations. The black solid line indicates the minimizer values, $x_{1}^{*}=0$ and $x_{2}^{*}=0$. (**c**) The maximum expected improvement remains about constant after the tenth iteration.

The global minimizer of this function is $x^{*}=\left[0,0\right]$. For this study, let us consider initial sampling plans with nine random designs, $X=\{x_{1},\ldots ,x_{9}\}$, as shown in Figure 4. Results summarized in Figure 5 show that in the case of 2D, the algorithm usually reaches convergence in about ten iterations.

## 4. Thermoplastic Starch Film Optimization

The proposed Bayesian experimental design is applied to the formulation of TPS film, aiming to optimize the plasticizer ($x_{1}$) and potato starch ($x_{2}$) weight percentages to maximize the film’s elongation at break ($y_{1}$) and tensile strength ($y_{2}$). Given the conflicting effects of plasticizer and starch content on the mechanical properties of TPS films and the difference in their noise effect, this application provides a template for tackling similar material optimization challenges.

### 4.1. Materials

The materials used in this study were sourced as follows: potato starch was obtained from Gefen Foods (Bayonne, NJ, USA); distilled white vinegar, 5 (m/v)% acetic acid, from Walmart’s Great Value (Bentonville, AR, USA); distilled water from Sigma-Aldrich (Burlington, MA, USA); and vegetable glycerol from Florida Laboratories (Fort Lauderdale, FL, USA).

Polymers are sourced from potato starch, and glycerol:vinegar mixture (in a 2:1 ratio) is utilized as the plasticizer. The acetic acid in the vinegar acts as an acid catalyst for facilitating the plasticization process [10]. Distilled water is used as a solvent to maintain a homogeneous mixture. It also acts as an additional plasticizing agent in the plasticization of TPS [49].

All ingredients utilized in this study adhere to the Food Chemical Codex (FCC) grade standards, ensuring their compostability. The TPS formation process outlined in Section 4.1 is designed to preserve the compostability characteristics of these ingredients and the TPS films.

### 4.2. Synthesis Protocol

The synthesis protocol for all experimental compositions is as follows:

**Mixing.** Wet ingredients (glycerol, vinegar, distilled water) are first combined in a 600 mL beaker. Potato starch is sifted using a 60-mesh sieve to ensure uniform granules, and is added while stirring continuously to achieve a homogeneous mixture. For all experimental compositions, the total initial mass of the mix is maintained at 120 g.

**Gelatinization.** The beaker containing the solution is placed on an electric hot plate and brought to the gelatinization temperature at around 80 °C at a heating rate of about 10 °C/min while stirring consistently. At the gelatinization point, the TPS color changes from milky to colorless and becomes more viscous. At this point, the beaker is taken off heat and cooled to about 60 °C.

**Figure 6:**
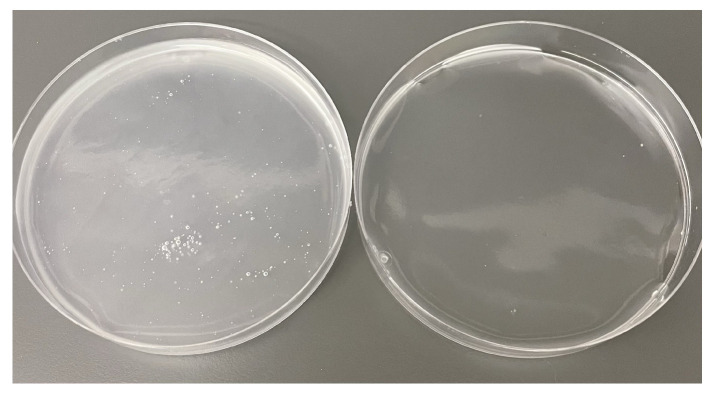
Centrifuging of TPS slurry effectively degases solution before air drying, leading to higher quality film. **Left**: 12 g of TPS slurry in a Petri dish without centrifuging. The solution is cloudy with many air bubbles. **Right**: 12 g of TPS slurry in a Petri dish after being centrifuged for two minutes at 2000 rpm. The solution is degassed and translucent.

**Degassing.** The TPS mixture is then centrifuged for two minutes at 2000 rpm to remove bubbles (Figure 6). The remaining mass of the TPS solution usually ranges between 72 and 78 g at this point.

**Drying.** The TPS mixture is poured into Petri dishes (12 g per Petri dish) and left to air dry in controlled laboratory ventilated air at room temperature (20 to 23 °C) for 72 h.

**Film preparation.** Finally, the dried film is detached from the Petri dish, and testing specimens are prepared for mechanical testing (Section 4.3).

### 4.3. Tensile Testing

TPS film samples were prepared to fit coupon-sized rectangles with a nominal dimension of 88 mm long by 13 mm wide, according to ASTM standard D638 [50]. Film thickness was measured with a Mitutoyo Digimatic digital micrometer with a 0.5 μm resolution. The specimen’s width was measured using a Mitutoyo Digimatic digital caliper with a 0.01 mm resolution. Three measurements were taken for each film sample and then averaged to estimate the film’s cross-sectional area. Tensile strength and elongation at break were determined by tensile testing on a universal testing machine (UTM) from Jinan Focus Test Instrument Co., Ltd. (Jinan City, Shandong, China). Five samples per formulation were tested, EB and TS were recorded, and the mean and variance of each property were determined.

### 4.4. Optimization

The iterative steps of the BO process, summarized in Figure 2, are the following:

**Initial sampling plan.** The initial sampling plan used in this work follows a full-factorial design of experiments (DOE) with two factors, $x_{1}$ and $x_{2}$, each at three levels, resulting in a sampling plan of nine points.

**Update exploration region.** The initial size of the exploration is 1.5 times larger than the initial DOE grid. The exploration region is updated dynamically, and when new experimental data are added, the region boundaries are adjusted based on the new sampling plan (Section 3.2).

**Observations.** One observation corresponds to the evaluation of a mechanical property (Section 4.3). After each new experiment, the observed data for the EB and TS properties, $y_{1}$ and $y_{2}$, and corresponding compositions, $x_{1}$ and $x_{2}$, are recorded. These observations provide the data to train the HGP model.

**HGP model.** Two GP models are trained for each property ($y_{1}$ and $y_{2}$) for a total of four GP models: $\mathrm{mean}(Y_{1})$, $\mathrm{var}(Y_{1})$, $\mathrm{mean}(Y_{2})$, and $\mathrm{var}(Y_{2})$. Each GP model has two outputs: mean and variance; therefore, the total number of outputs of the HGP model is eight: $\mathrm{mean}(\mathrm{mean}\left(y_{1}\right))$, $\mathrm{var}(\mathrm{mean}\left(y_{1}\right))$, $\mathrm{mean}(\mathrm{var}\left(y_{1}\right))$, $\mathrm{var}(\mathrm{var}\left(y_{1}\right))$, $\mathrm{mean}(\mathrm{mean}\left(y_{2}\right))$, $\mathrm{var}(\mathrm{mean}\left(y_{2}\right))$, $\mathrm{mean}(\mathrm{var}\left(y_{2}\right))$, and $\mathrm{var}(\mathrm{var}\left(y_{2}\right))$. The HGP model updates continuously with new experimental results, refining the predictions and uncertainty quantification predictions and providing data to the acquisition functions.

**Acquisition function.** The acquisition functions, LCB (25) and EI (28), guide the selection of new sampling points based on the HGP model’s outputs. In each iteration, four new points are located: one from EI and one from LCB point for each targeted mechanical property. The four corners of the exploration region Γ are evaluated to promote broader exploration. While up to eight distinct points can be added per iteration, in practice, some points may overlap or already be included in the sampling plan, resulting in fewer new additions.

**Convergence.** The convergence criteria are satisfied when there is minimal improvement in material properties over successive iterations or when a predefined number of experiments is met (Section 3.3). At the point of convergence, an optimal or near-optimal solution within the explored region has been found. If the convergence is reached, the optimal design corresponds to $x_{\mathrm{best}}$. This application will have one optimal design for each material property $y_{1}$ and $y_{2}$.

**Update sampling plan.** If the convergence criteria are not satisfied, the new designs are tested, and the sampling plan is updated. The BO process iterates until a convergence criterion is met.

## 5. Results and Discussion

**Figure 7:**
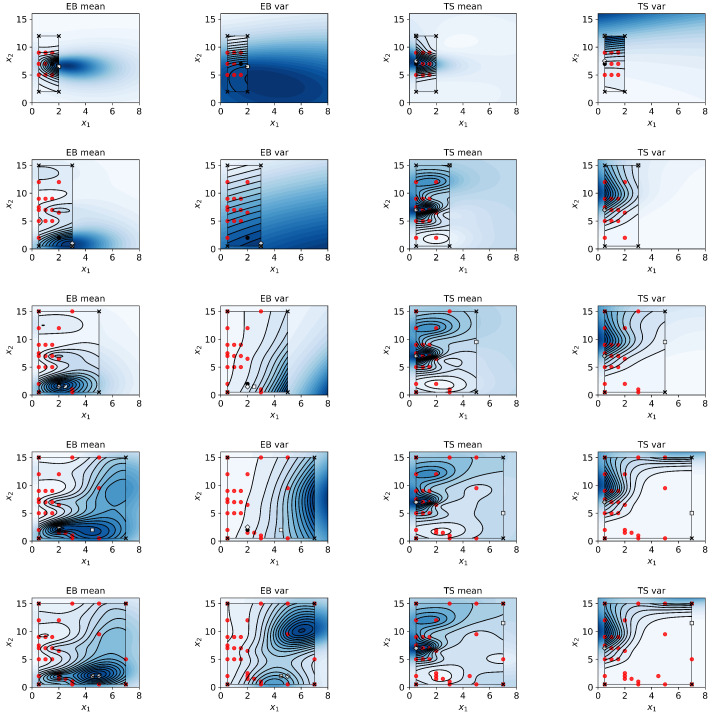
Results from HGP predictions from TPS DOEs for five experimental iterations. As in the numerical experiments, an initial design was evaluated, and an exploration region was initialized. BO guided the subsequent experiments until formulations yielding optimal mechanical properties were achieved. The red dots represent the design points evaluated in each iteration. HGP models were used to predict the mean and variance of EB and TS for varying compositions of plasticizer ($x_{1}$) and starch content ($x_{2}$). The far left column displays HGP predictions for EB, with the second column showing the predicted variance for EB. The next column shows the mean TS predictions, with the final column showing the predicted variance for TS.

**Figure 8:**
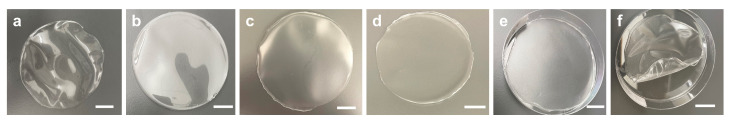
TPS film: Increased starch content with decreased plasticizer content to decreased starch content with increased plasticizer content. TPS film with increased and decreased starch content led to brittleness and warping. Similarly, decreased starch and increased plasticizer content also caused film warping and moisture. (**a**) The film specimen with the highest starch content and least plasticizer content. The film is warped and extremely brittle, with low mechanical properties. (**b**) The film specimen with increased plasticizer content. The film is less brittle and exhibits optimal TS. (**c**) The film specimen has roughly equal parts of plasticizer and starch content. Mechanical properties are suitable for both EB and TS. (**d**) The film specimen with slightly more plasticizer than starch. EB is increased, but subsequently, the TS begins to diminish. (**e**) Increased plasticizer leads to slight warping, films retain more moisture and exhibit optimal EB, and diminished TS. (**f**) Film specimen with the highest plasticizer concentration and the lowest starch concentration. Films are extremely deformed and moist. TS is diminished, and EB begins to decrease with worsening film quality.

**Figure 9:**
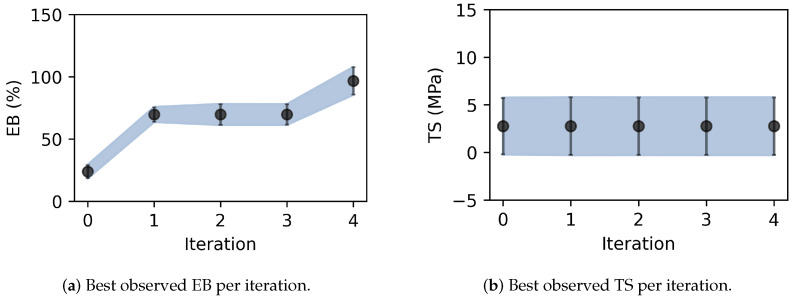
Best observed properties. The black dots represent the mean best-observed property per experimental observation. The vertical bar and blue-shaded region indicate the 95% confidence interval. (**a**) The optimal formulation for maximizing EB was found after four iterations, with increased plasticizer concentrations leading to greater EB variability. (**b**)The optimal formulation for TS was found after the first iteration, with relatively constant variability.

Five experimental iterations were evaluated. Each iteration takes roughly one week to complete. The TPS elongation at break (EB) and tensile strength (TS) are affected by the plasticizer (glycerol:vinegar) ($x_{1}$) and starch content ($x_{2}$) in a nonlinear manner (Figure 7). Specifically, higher content of plasticizer (up to about 4.5 wt%) and low starch content (down to about 2.0 wt%) maximize EB. In contrast, low content of plasticizer (down to about 0.5 wt%) and medium-level content of starch (about 7.0 wt%) maximize TS. Notably, an excessive amount of plasticizer (above 6.0 wt%) or excessive amount of starch (above 12.0 wt%) has a negative effect on the overall structural stability of the film and its mechanical properties, leading to increased film brittleness and decreased EB. Figure 8 displays the variations in film quality with varying amounts of starch and plasticizer content. Furthermore, our approach allows quantifying the uncertainty of the response as a function of the material content (Figure 9). For EB, higher variance is observed in specimens with higher content of both plasticizer and starch. The variance function is more flat for TS but increases with the starch content.

As summarized in Table 1, the optimal formulation for the highest EB was found in the last iteration to be 4.5 wt% plasticizer, 2.0 wt% starch, and 93.5 wt% water (Figure 9). The corresponding optimal TPS contains 3.6 g glycerol, 1.8 g vinegar, 2.4 g starch, and 112.2 g water. Compared to the EB value of about 11% to 31% reported in previous studies [9,10], this composition leads to a higher EB value (M = 96.7%, SD = 5.6%), which represents an increase of about 212%.

On the other hand, the optimal formulation for the highest TS was found in the first experimental iteration to be 0.5 wt% plasticizer, 7.0 wt% starch, and 93.5 wt% water (Figure 9 and Table 2). The corresponding optimal TPS contains 0.4 g glycerol, 0.2 g vinegar, 8.4 g starch, and 111.0 g water. Compared to the TS value of about 1.03 MPa to 2.04 MPa from previous studies [9,10], this formulation leads to around a higher TTS value (M = 2.77 MPa, SD = 1.54 MPa), which represents a 38% increase.

Plasticizing agents, extracted starch, and acid catalysts significantly affect the mechanical, structural, and physical properties of TPS films [18]. Additional factors, such as the time and temperature of film preparation, may also affect the material’s mechanical properties. Previous studies have investigated the effects of various plasticizing agents such as choline chloride and urea [10], xylitol and sorbitol [51], and glycerol-sorbitol [16]. The most commonly utilized plasticizer in the fabrication of TPS films is glycerol/water [49]. The presence of low-weight amide plasticizing structures in the addition of water, such as glycerol, has been shown to perform significantly better at suppressing starch retrogradation as opposed to other plasticizing agents [17]. Additionally, other low-weight organic acids such as acetic acid or citric acid [52] further contribute to suppressing starch reintegration, effectively reducing the material’s degradation rate while also acting as a catalyst for plasticization [53].

Increased amounts of glycerol in relation to starch tend to amplify variations in the observed mechanical properties of TPS film [54]. This is most likely explained by changes in the starch’s crystalline structure post-retrogradation. Studies such as those by Paluch et al. [55] and Surendren et al. [6] highlight that the addition of plasticizers disrupts the natural crystalline structure of starch, leading to a higher proportion of amorphous regions. This structural transformation results in increased chain mobility and free volume within the polymer matrix, which promotes more efficient starch gelatinization and flexibility. However, this increase in flexibility is accompanied by a reduction in material strength since the crystalline regions responsible for stiffness are diminished by the plasticizer-induced amorphous structures.

Since amorphous regions are more flexible and less rigid than crystalline regions, the overall mechanical properties (such as tensile strength and elongation at break) of the material become more sensitive to small changes in the glycerol concentration. With more glycerol, the proportion of amorphous regions in the starch increases while the amount of crystalline regions decreases. This sensitivity contributes to greater variability in the observed properties of TPS film because the balance between amorphous and crystalline regions can significantly affect the material’s properties. Starch content and the presence of amylose have little effect on TS because the overall balance of crystalline and amorphous regions in TPS is more dependent on plasticizer content and is not dramatically altered by varying starch content [56,57]. On the contrary, increased amounts of starch content are found to decrease the EB and, in large concentrations, contribute to severe film brittleness [56,58].

The trade-off between TS and EB must be carefully managed depending on the desired application of the TPS film by carefully identifying optimal starch and plasticizer concentrations. Plasticizers like glycerol disrupt the intermolecular hydrogen bonding between starch molecules, leading to a more amorphous and flexible material, but at the cost of TS. While starch content may not significantly influence the mechanical properties of TPS due to its limited effect on the overall crystalline-amorphous balance, plasticizers weaken these interactions, resulting in lower TS values across most TPS formulations. Increasing plasticizer content leads to softer, more deformable films with reduced tensile strength and increased EB; however, it contributes to more variation in the observed properties. Increased starch content results in a reduction of EB, with little impact on the TS; however, it also leads to increased film brittleness. A balance between plasticizer and starch content is needed to maintain satisfactory film quality free of warping, brittleness, or excess moisture retention that may negatively impact the mechanical properties.

## 6. Conclusions

This study presents a novel Bayesian optimization approach for maximizing the elongation at break and tensile strength of TPS film by varying the plasticizer and starch content. The proposed framework addresses the challenges of DOE methodologies and traditional Bayesian approaches, including inefficiency, handling of heteroscedastic noise, and exploration region management. The proposed Bayesian approach leverages an HGP model suitable for physical experiments. This model independently predicts the mean and variance of the material properties, allowing for more accurate predictions of non-uniform noise. The optimization process is guided by two acquisition functions (LCB and EI) and a dynamic exploration strategy to adjust the design space boundaries. Validation via numerical benchmark tests, including the Forrester’s and three-hump camel functions, demonstrated the framework’s capability to efficiently handle noise in multidimensional optimization problems. When applied to TPS films, the approach iteratively achieved EB and TS improvements, highlighting its potential to accelerate material optimization and enhance sustainable material performance.

This work’s main contributions are as follows: First, it introduces a practical HGP model for Bayesian experimental design built from traditional GP models. The HGP implementation allows the use of established modeling tools with minimal modifications. Second, it presents an adaptive exploration scheme that systematically expands the design space by managing the exploration region. The two acquisition functions, along with the samples from the corner of the exploration region, enhance the diversity of candidate designs. In practice, this approach enables the generation of multiple designs per iteration, making it more feasible for physical experiments. Finally, this work pioneers a BO approach to formulate TPS films.

The results of this work are limited by the number and nature of design variables, the number of experiments, and the number of objectives. Only two variables are considered: plasticizer and starch content. Additional variables include additives, process parameters, or the relative molecular weight of TPS polymers through Gel permeation chromatography (GPC), which may positively affect the film’s mechanical properties. Additionally, this work is constrained by having only 30 total experiments. More experiments could refine the results and further validate the optimization framework. Finally, this study focused on optimizing two objectives: elongation at break and tensile strength. Expanding the optimization framework to include additional properties such as hydrophobicity or compostability could offer deeper insights. Moreover, further characterization that can be gathered from stress–strain curves such as Young’s modulus, yield strength, and strain hardening could provide a more comprehensive understanding of the material’s potential to replace traditional plastic films. Despite the limitations of this study, the proposed work serves as a template to optimize material systems more efficiently.

## Figures and Tables

**Table 1:** Summary of iterative results for elongation at break (EB). Results include the plasticizer and starch composition of the best design, the EB mean and standard deviation, the expected improvement at the end of the iteration, and the cumulative number of experiments.

Iter	Plast (wt%)	Starch (wt%)	EB (%)	EI	Exp
1	1.5	7.0	23.9 ± 2.7	6.06	9
2	2.0	2.0	69.8 ± 3.0	32.5	15
3	2.0	2.0	69.8 ± 4.2	2.97	20
4	2.0	2.0	69.8 ± 4.2	2.23	25
5	4.5	2.0	96.7 ± 5.6	3.16	30

**Table 2:** Summary of iterative results for tensile strength (TS). Results include the plasticizer and starch composition of the best design, the TS mean and standard deviation, the expected improvement at the end of the iteration, and the cumulative number of experiments.

Iter	Plast (wt%)	Starch (wt%)	TS (MPa)	EI	Exp
1	0.5	7.0	2.77 ± 1.51	0.615	9
2	0.5	7.0	2.77 ± 1.54	0.609	15
3	0.5	7.0	2.77 ± 1.54	0.605	20
4	0.5	7.0	2.77 ± 1.54	0.604	25
5	0.5	7.0	2.77 ± 1.54	0.604	30

## Data Availability

The original contributions presented in the study are included in the article. The raw data and the data analysis code are openly available on the following GitHub repository: https://github.com/andres-tovar-purdue/materials/, accessed on 26 October 2024. Further inquiries can be directed to the corresponding author.
